# Diversity of Parasitoid Wasps and Comparison of Sampling Strategies in Rice Fields Using Metabarcoding

**DOI:** 10.3390/insects15040228

**Published:** 2024-03-26

**Authors:** Liyang Wang, Hongxuan Wu, Wei He, Guihong Lai, Junxi Li, Siling Liu, Qiang Zhou

**Affiliations:** State Key Laboratory of Biocontrol, School of Life Sciences, Sun Yat-Sen University, Guangzhou 510275, China; wangly96@mail2.sysu.edu.cn (L.W.); wuhx69@mail2.sysu.edu.cn (H.W.); hewei83@mail2.sysu.edu.cn (W.H.); laigh6@mail2.sysu.edu.cn (G.L.); lijx385@mail2.sysu.edu.cn (J.L.); liusling7@mail2.sysu.edu.cn (S.L.)

**Keywords:** metabarcoding, biodiversity, rice fields, parasitoid wasps

## Abstract

**Simple Summary:**

DNA metabarcoding was used to survey the biodiversity of parasitoid wasps in rice fields. DNA was collected from tissue samples of selected parasitoid wasps in the ethanol of Malaise traps. The results demonstrated the utility of the ethanol filter method for obtaining species information. However, it may lack detailed information and potentially lead to a reduced estimate of species richness. We also identified additional insect species in the parasitoid samples through metabarcoding. The results demonstrate the efficacy of high throughput sequencing on adult parasitoid wasps to determine their host associations. These data enhance the understanding of host species and provide insights into food web dynamics.

**Abstract:**

A comprehensive and precise evaluation of Arthropoda diversity in agricultural landscapes can enhance biological pest control strategies. We used Malaise traps and sweep nets to collect insects from three double-cropping paddy fields. DNA was extracted from the ethanol preservative of the Malaise traps and from tissue samples of selected parasitoid wasps. This was followed by amplification using DNA barcoding primers to prepare high-throughput sequencing libraries. We annotated a total of 4956 operational taxonomic units (OTUs), encompassing 174 genera and 32 families of parasitoid wasps. The ethanol filter method efficiently captured a wide range of information. However, the method has low resolution and may result in a reduced estimate of species abundance. Additional insect species were also identified in the parasitoid samples. This suggests that high throughput sequencing from adult parasitoid wasps can also detect host species, enabling a better understanding of host species and providing insights into food webs.

## 1. Introduction

The stability and health of natural enemy communities helps maintain the sustainability of farmland ecosystems and agricultural production [1,2]. The diversity of parasitoid wasp communities in rice fields contributes to the level of pest control [1]. Many parasitoid wasps, such as species in the Trichogrammatidae, Braconidae, Dryinidae, and Mymaridae families, help control agricultural pests such as *Cnaphalocrocis medinalis* Guenee (Lepidoptera: Pyralidae), *Nilaparvata lugens* (Stål) (Hemiptera: Delphacidae), and *Sogatella furcifera* (Horváth) (Hemiptera: Delphacidae) [3,4,5]. However, pest management measures, seasonal changes and environmental factors affect the balance between rice pests and their natural enemies, and the diversity of parasitoids constantly varies [6,7,8]. In the subtropical smallholder agroecosystems of typical rice, vegetables, and sugarcane in southern China, the insect diversity of different types of vegetation strips has been studied. Pesticides, ridge vegetation, and crop heterogeneity have significant effects on the diversity of beneficial insects [9,10,11]. Therefore, it is important to monitor the diversity of natural enemies to help optimize their value.

There is rich diversity of parasitoid wasps within the major rice ecosystems across Asia with approximately 240 species being documented. Among these, 65 species parasitize Hemiptera species, while 145 species target Lepidoptera [12,13]. A recent survey documented 109 species of parasitoid wasps associated with Hemiptera in rice fields. The highest species richness was found in the Mymaridae (31 species), followed by the Dryinidae (21 species) [14]. The small size and morphological similarities among closely related and cryptic species necessitate the use of taxonomic experts for accurate identification. Taxonomic classification and identification based on morphological traits, including external morphology and genitalia, requires substantial expertise, time, and resources [15,16]. Due to these limitations, some studies have only identified specimens to genus and family. Therefore, an alternate method is needed that facilitates more rapid and accurate species identification.

Sequencing technology has enabled the utilization of metabarcoding, which integrates barcoding and high-throughput sequencing. This technology has become increasingly prevalent in species diversity survey research [17,18]. High-throughput sequencing, in contrast to the Sanger sequencing method, offers the advantage of heightened sensitivity of first-generation sequencing. It also enables the rapid identification of numerous species using relatively small-sequenced fragments. This mitigates instances where target sequences fail to amplify due to DNA degradation. The amplification range is wide, and the universal primers using DNA barcode are suitable for most insects [19].

Little research has been conducted on the diversity of parasitoids in rice fields using molecular identification methods. Existing studies primarily rely on the first-generation sequencing of the COI gene to identify a small subset of parasitoids [20]. However, there is much less research focusing on the diversity of parasitoids in rice fields utilizing high-throughput second-generation sequencing via metabarcoding technology. Further investigation is required to understand the diversity of parasitoids in rice fields and the use of molecular techniques for accurate identification.

The extraction of samples for metabarcoding sequencing typically involves two different methods. One involves the destructive method of tissue grinding, while the other involves the non-destructive method of extracting DNA from the sample preservation solution [21]. The destructive method of tissue grinding can provide a large amount of DNA but causes sample destruction and the loss of morphological characteristics [22,23]. The extraction of DNA from the ethanol of preserved samples is a common method [12]. Ethanol extraction is fast, and the ability to distinguish different species is comparable to other sample processing methods [24]. However, it has been reported that the DNA extracted from ethanol only recovers 15.9% of the genera and 11.2% of the families identified by morphological classification [25]. This would represent a significant information loss. The objective of the present study was to evaluate information and methodologies for the rapid surveillance of the species diversity of parasitoid wasps in rice fields. This was achieved by the analysis and comparison of data acquired using two DNA extraction methods. From 2022 to 2023, Malaise traps and sweep nets were used to collect insects from double-cropping rice fields with different management modes. DNA was directly extracted from the ethanol of the preserved parasitoid wasp samples, followed by subsequent extraction by grinding the samples. The universal primers of the DNA barcode region were used for amplification and a sequencing library was prepared using the Illumina platform for high-throughput sequencing.

## 2. Materials and Methods

### 2.1. Sample Collection and Storage

The specimens were collected from rice paddy fields at the Wushan farm of the South China Agricultural University (WS) (23.162239° N, 113.360629° E), Conghua district (CH) (23.552548° N, 113.583478° E) and Zengcheng district (ZC) (23.275373° N, 113.699062° E) in Guangdong Province, China, from March 2022 to November 2023.

The WS site is characterized by cement structures with limited vegetation cover and frequent insecticide applications. The CH site features abundant vegetation with diverse flowering plants and no insecticide usage. The ZC sample site has moderate vegetation cover and infrequent insecticide applications.

Samples were collected using sweep nets and Malaise traps. Five sampling areas were randomly established in each location. Each sample area was swept 50 times along a 100 m ridge with an insect net (handle length: 100 cm; net diameter: 38 cm). This approach ensured robust sampling by forcefully sweeping the net through the rice canopy [26,27]. The collecting jars of the Malaise traps were replaced monthly with new jars filled with 95% ethanol.

All sweep net and Malaise trap samples from each location during one rice cropping season were combined. The early rice cropping season spanned from March to June, while the late rice cropping season extended from August to November. The pooling resulted in a total of 12 samples. The collected insect samples were stored in 95% ethanol at −20 °C in the laboratory.

### 2.2. DNA Extraction and Amplification

All of the preservative ethanol from samples was filtered through 0.45 μm nitrocellulose filters connected to a gas–vacuum pump [28]. The filters were then fragmented into small pieces, using fine tweezers, and dried. DNA extraction from the filters was performed with the DNeasy Blood and Tissue Kit (QIAGEN, Hilden, Germany) following the manufacturer’s instructions. Insect samples were immersed in new 95% ethanol and stored at −20 °C until processed.

One hundred parasitoid wasps were randomly taken from each sample. The parasitoid wasps from each site were combined and divided into two samples. Each sample was homogenized by grinding the tissue using a sterilized electrical grinding rod. DNA extraction was performed according to manufacturer’s instructions, as described above. The success of extraction and DNA quality was assessed using 1% agarose gels.

PCR amplification was performed using the barcode universal primer mlCOlintF-jgHCO2198 [29], which is suitable for high-throughput sequencing, to amplify mitochondrial COI gene fragments that are about 300 bp in length. PCR was performed with Taq DNA Polymerase (Takara Bio Inc., Kusatsu, Japan). The 50 μL PCR reaction mixture contained 0.25 μL of rTaq DNA polymerase (5 U/μL), 5 μL of 10× PCR Buffer (Mg^2+^, plus), dNTP mixture 4 μL, 2 μL F primer (10 μM), 2 μL R primer (10 μM), 5 μL of DNA, and 35.75 μL of water. The PCR cycling conditions were 94 °C for 1 min, 35 cycles of 98 °C for 10 s, 55 °C for 30 s, 72 °C for 30 s, and finally, 72 °C for 5 min [29]. The PCR products were utilized for NGS sequencing.

### 2.3. High Throughput Sequencing and Data Analyses

Purified PCR products were quantified by Qubit 3.0 (Life Invitrogen, Carlsbad, CA, USA; Thermo, Waltham, MA, USA). The pooled DNA product was used to construct an Illumina pair-end library following the Illumina genomic DNA library preparation procedure. Then, the amplicon library was pair-end-sequenced (2 × 250) on an Illumina MiSeq platform (Shanghai BIOZERON Co., Ltd., Shanghai, China) using standard protocols.

Raw fastq files were first demultiplexed using in-house perl scripts according to the barcode sequences information for each sample with the following criteria: (i) The 250 bp reads were truncated at any site, receiving an average quality score < 20 over a 10 bp sliding window and discarding the truncated reads that were shorter than 50 bp. (ii) Exact barcode matching, two nucleotide mismatches in primer matching, and reads containing ambiguous characters were removed. (iii) Only sequences with overlaps longer than 10 bp were assembled according to their overlap sequence. Reads that could not be assembled were discarded.

Operational taxonomic unit (OTU) clustering was conducted according to 97% similarity; the OTUs were obtained by removing chimera and repeat sequences.

The diversity index was calculated using mothur software version 1.22 [30], including the Chao, ACE, Simpson, and Shannon diversity indices. The coverage of this sequencing was calculated by subtracting the ratio of the number of OTUs containing only one sequence and the number of all sequences. Evenness is the ratio of the actual Shannon index of the community to the maximum Shannon index that can be obtained in a community with the same species richness. Richness is the total number of species whose abundance is greater than 0 in the community, and the larger the value, the richer the species in the community.

The OTU abundance table of parasitoid wasps from all samples was analyzed by principal coordinate analysis, the distance between samples was calculated using Bray–Curtis dissimilarity [31], and the confidence ellipse was drawn with a confidence level of 0.95.

## 3. Results

### 3.1. OTU Taxonomic Assignment

**Figure 1:**
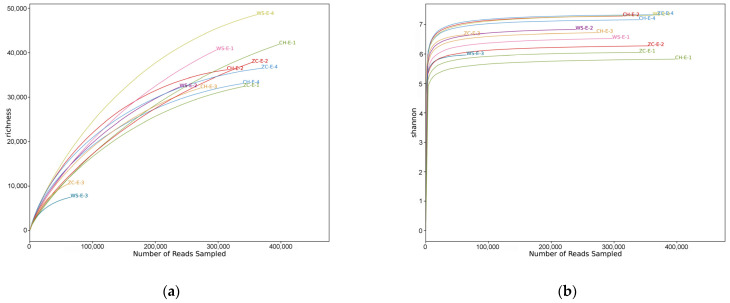

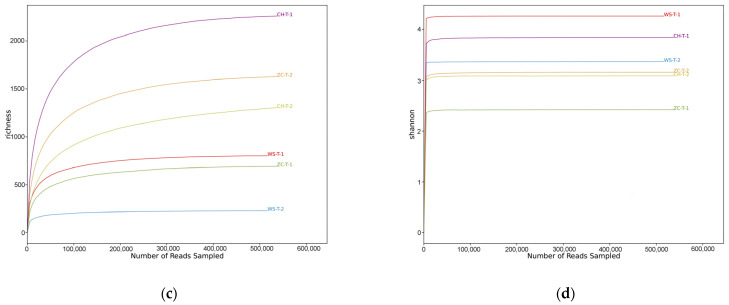
(**a**) Rarefaction curve of ethanol samples; (**b**) Shannon–Wiener curve of ethanol samples; (**c**) rarefaction curve of tissue samples; (**d**) Shannon–Wiener curve of tissue samples. The name of each sample is indicated alongside its corresponding curve, representing the sampling location, DNA extraction methods, and serial number. CH: Conghua district; ZC: Zengcheng district; WS: Wushan farm. E: ethanol samples; T: tissue samples. The corresponding grouping information is presented in Table S1.

- Ethanol samples: After splicing and removing impurities, 7,391,894 sequences were obtained by high-throughput sequencing, with a total of 2,311,835,324 bp and an average fragment size of 312.753 bp. A total of 243,783 OTUs were obtained by removing chimera and repeat sequences. The OTU abundance in the 12 samples was flattened out for subsequent analysis. The coverage of 12 samples was between 0.93–0.99 (Table S1), indicating that the depth of sequencing was reasonable and fully reflected the richness of samples. Both the rarefaction curve (Figure 1a) and Shannon–Wiener curve (Figure 1b) tended to be flat, indicating that the sequencing data were large enough to reflect most of the biological information in the sample.
- Tissue samples: A total of 2,444,490 sequences were obtained by high-throughput sequencing with a total of 745,281,495 bp and an average fragment size of 304.88 bp. A total of 4446 OTUs were obtained. The OTU abundance of four samples was flattened out for subsequent analysis. The coverage of four samples was greater than 0.999 (Table S1), indicating that the depth of sequencing was reasonable and fully reflected the richness of samples. Both the rarefaction curve (Figure 1c) and Shannon–Wiener curve (Figure 1d) tended to be flat.

### 3.2. Species Composition Analysis

**Figure 2:**
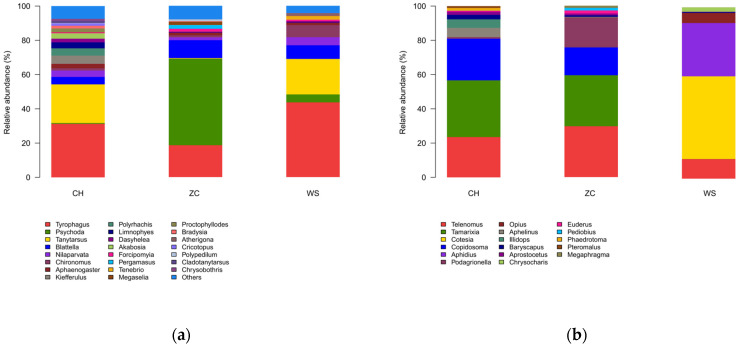
Species community bar plot in genus level of two methods from three locations (**a**) ethanol samples; (**b**) tissue samples. CH: Conghua district; WS: Wushan farm; ZC: Zengcheng district. Different colors represent different genera. The taxonomic information is presented in Table S2. The percentage of each taxonomic level is provided in Table S3.

From the ethanol samples, 243,783 OTUs were recorded as 9 classes, 35 orders, 320 families, 1358 genera, and 2796 species of arthropods (Figure 2a). A total of 576 species in 9 orders, 89 families, and 243 genera were identified in the tissue samples (Figure 2b).

Among the ethanol samples, species of Diptera exhibited the highest abundance, comprising 48% of all OTUs, followed by Sarcoptiformes (31%), Blattodea (7%), Hymenoptera (4%), and Hemiptera (3%) (Figure 2a). Diptera also exhibited the highest species richness with 1778 species, while Lepidoptera had 300 species, Coleoptera had 243 species, and Hemiptera had 73 species. Hymenoptera were the most prevalent taxon among the tissue samples with a frequency of 98%, whereas Diptera and Lepidoptera annotations accounted for less than one percent (Figure 2b, Table S3).

**Figure 3:**
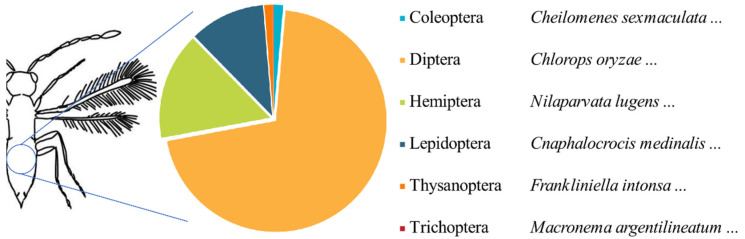
Other insects detected in tissue samples of parasitoid wasps.

Several important agricultural pests, including *Cnaphalocrocis medinalis* Guenee (Lepidoptera: Pyralidae), *Chlorops oryzae* Matsumura (Diptera: Chloropidae), *Nilaparvata lugens* (Stål) (Hemiptera: Delphacidae), and *Sogatella furcifera* (Horváth) (Hemiptera: Delphacidae) were detected from the tissue samples of parasitoid wasps (Figure 3).

### 3.3. Group Comparison Analyses

**Figure 4:**
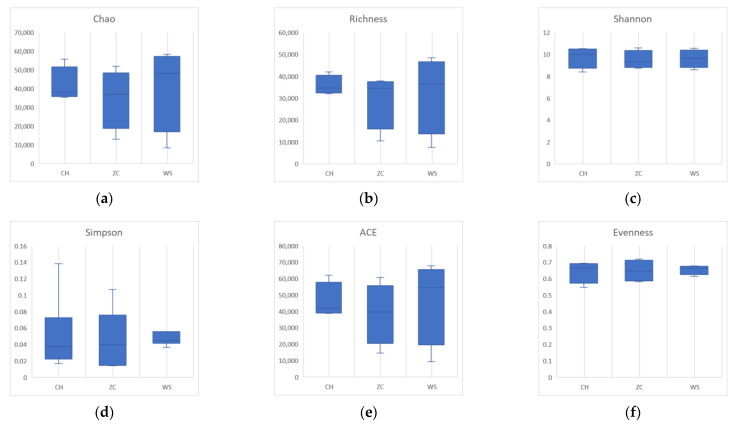
Alpha diversity estimates boxplots of ethanol samples from three locations. (**a**) Chao1; (**b**) richness; (**c**) Shannon index; (**d**) Simpson index; (**e**) ACE (abundance-based coverage estimator) index; (**f**) evenness. CH: Conghua district; WS: Wushan farm; ZC: Zengcheng district.

**Figure 5:**
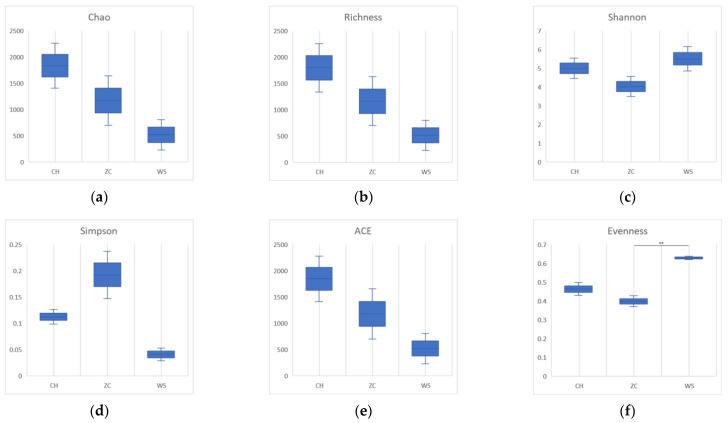
Alpha diversity estimates boxplots of tissue samples from three locations. (**a**) Chao1; (**b**) richness; (**c**) Shannon index; (**d**) Simpson index; (**e**) ACE (abundance-based coverage estimator) index; (**f**) evenness. CH: Conghua district; WS: Wushan farm; ZC: Zengcheng district. The short line represents the significant difference between different locations calculated using Tukey’s multiple comparisons test. The asterisks represent the degree of difference: ** *p* < 0.01.

All samples were grouped and compared according to the collection site and the two DNA extraction methods. In the ethanol samples, there were no significant differences in species richness, diversity, or evenness (*p* > 0.05) (Figure 4). The community richness calculated by the Chao and Richness index of CH was higher than that of the other two groups in tissue samples. The community diversity calculated by the Shannon and Simpson indices was higher in WS. The evenness of WS was significantly higher than that of ZC (*p* = 0.0083) (Figure 5).

### 3.4. Differences in Species Diversity of Parasitoid Wasps Noted in the Two Treatments

**Figure 6:**
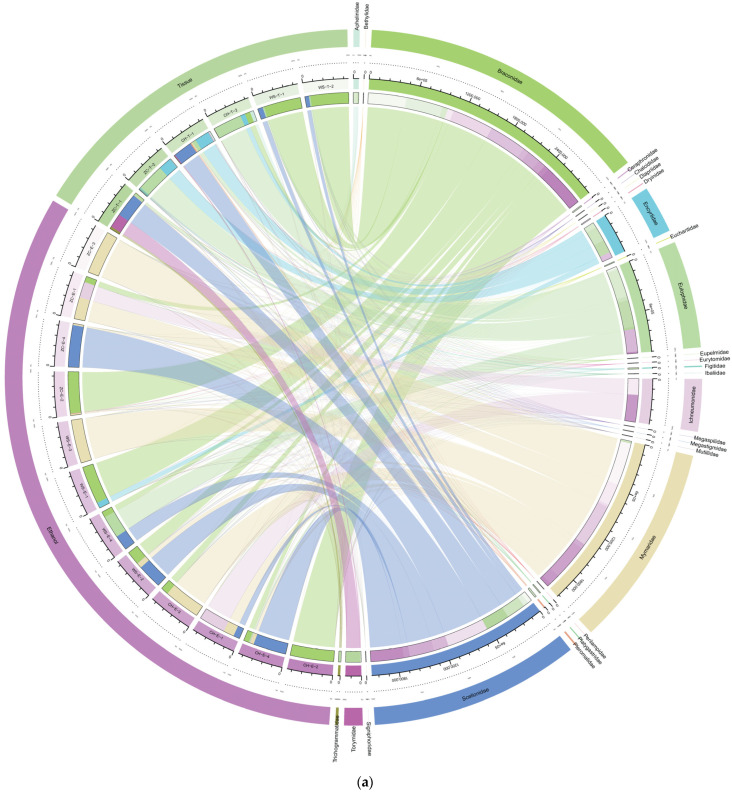

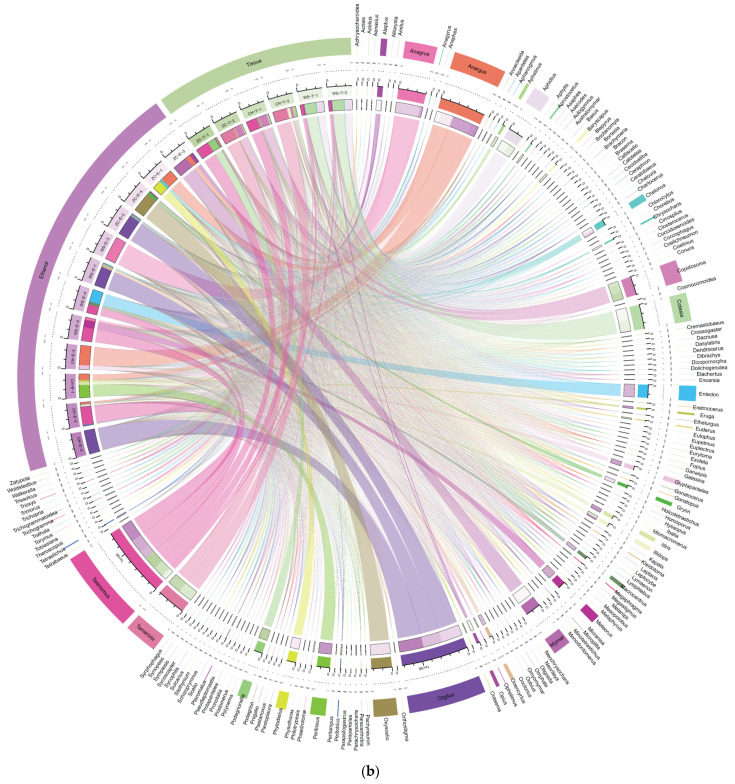
Diversity of parasitoid wasps in all samples. (**a**) Family-level parasitoid wasp diversity; (**b**) genus-level parasitoid wasp diversity. Outermost circle: the sample grouping information circle; interior circle: the relative abundance percentage circle; inner line: the out-sample association line. The left side of the circle represents samples, the right side represents taxa, different colors represent different taxa and sample groups.

All OTUs annotated to the parasitoid part of Hymenoptera were flattened out and grouped according to two sample processing methods, the tissue sample and the ethanol sample, for comparison. A total of 748 OTUs of 7 families and 31 genera of parasitoids were annotated from all the ethanol samples, while 4208 OTUs of 143 genera of 25 families were annotated from the tissue samples. The ethanol samples lost at least 72% of the families (Figure 6a), 78% of the genera (Figure 6b), and 82.2% of the OTUs. The species diversity of parasitoids in tissue samples was much higher than the diversity in ethanol samples.

The highest abundance in ethanol samples was Braconidae (69%), followed by Mymaridae (17%), Scelionidae (10%), and others (4%) (Figure 6a). The most species-rich families were Braconidae (16 species), followed by Scelionidae (11 species) and Mymaridae (9 species).

In tissue samples, the highest abundance was Eulophidae (35%), followed by Scelionidae (25%), Encyrtidae (18%), and Braconidae (3%). Mymaridae and Trichogrammatidae accounted for 1% (Figure 6a). There were 92 species belonging to Eulophidae, 79 species belonging to Scelionidae, 63 species belonging to Braconidae, 61 species belonging to Mymaridae, 36 species belonging to Pteromalidae, and 29 species belonging to Trichogrammatidae.

**Figure 7:**
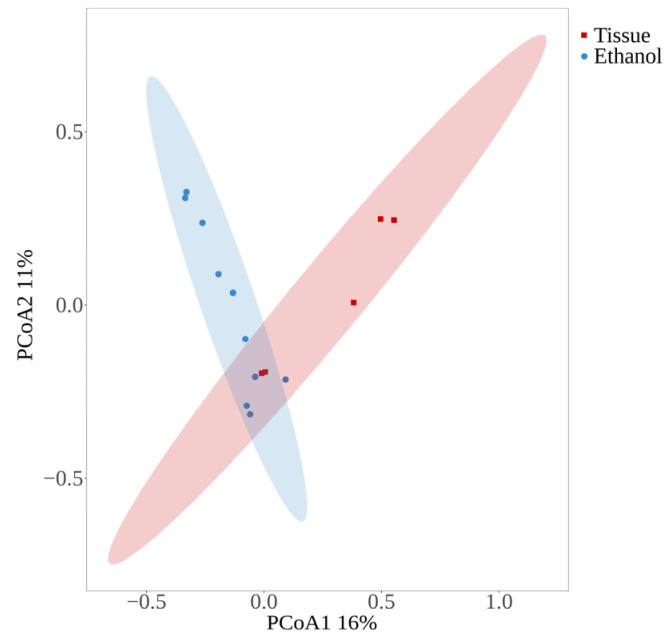
Principal coordinates analysis (PCoA) of parasitoid OTUs of two sample handling methods; plots show PCoA1 and PCoA2. The confidence ellipse was drawn with a confidence level of 0.95.

The sample confidence ellipses of both groups were not significantly separated (Figure 7), but the projections on different principal components were significantly different, with PC1 separating samples from different sample processing methods. PC1 can explain 16% of the variation between samples, PC2 can explain 11% of the variation between samples, and PC3 can explain 9% of the variation between samples.

## 4. Discussion

### 4.1. Diversity of Parasitoid Wasp Communities

The tissue samples of parasitoid wasps from the three sites exhibited significant differences in evenness (*p* = 0.0083). However, no significant differences were observed when comparing diversity indices calculated from ethanol samples of the three sample sites (*p* = 0.5747). This suggests that sampling methods can greatly influence survey results. Therefore, the careful selection of appropriate sampling methods is crucial for the accurate assessment of biodiversity.

Based on the second-generation sequencing results obtained from the two sampling methods, a total of 4956 OTUs belonging to 174 genera and 32 families of parasitoid wasps were annotated. This number is much larger than the previously reported 240 species [12,13,32], indicating that the current assessment of parasitoid wasp species diversity in paddy fields is underestimated.

### 4.2. Limitations of Ethanol DNA Extraction Methods

We found that the use of ethanol filtration enables the rapid and extensive acquisition of biological information from Malaise trap samples. However, it reduces resolution at specific taxonomic levels. A total of 748 OTUs belonging to 7 families and 31 genera were identified across all combined samples using the ethanol extraction method. Comparatively, the grinding tissue method significantly improved sequencing resolution, resulting in the annotation of 4208 OTUs from 143 genera within 25 families. Both the rarefaction curve and the Shannon–Wiener curve indicated sufficient sequencing depth for the entire sample set. It is likely that the low DNA content per insect in the filtration method leads to the loss of many non-dominant species during subsequent amplification and sequencing steps. DNA extraction through selective grinding allows researchers to achieve higher overall resolution and obtain more detailed information by targeting specific taxonomic levels within arthropod communities.

A diversity analysis of arthropod samples collected in Richmond Park, Surrey, UK, yielded similar results. In this study, only 40% of the species were recovered from ethanol samples compared to 92% from tissue samples [33]. A study conducted in Stockholm, Sweden, examined the metabarcoding diversity of Malaise trap tissue and ethanol and found significant variation in estimates of community composition [34]. In addition, other research has demonstrated that ethanol extraction can result in reduced species compositions when compared to tissue grinding [28].

### 4.3. Species Annotation and Abundance

Considerable molecular information was obtained in this study. Compared to traditional classification methods and DNA barcoding studies by generation sequencing, the high-throughput sequencing used here is cheaper and can quickly obtain all DNA barcoding information from samples. Maximum cost saving depends on the quantity of the samples [35].

Species annotation: There are differences in the intraspecific variations of barcode sequences within different taxonomic groups. For example, certain COI sequences in *Chortoicetes terminifera* exhibit intraspecific differences ranging from 2 to 6% [36]. Hawaiian *Hylaeus* bee species exhibit intraspecific differences of 4% [37]. Mitochondrial heterogeneity can potentially complicate species identification by clustering sequences from the same species into OTUs and annotating them as distinct species. This may lead to the overestimation of species diversity. To ensure accurate classification in macro-barcoding studies, it is crucial to rely on a well-curated reference database that associates DNA marker sequences with morphologically verified specimens [38]. In this study, annotations were made using the NCBI and BOLD databases [39]. In the present study, the species delimitation analysis was clustered into OTUs using the Deblur denoising algorithm. However, there are more useful methods, like distance-based (ABGD and jMOTU threshold analysis) and tree-based (GMYC and PTP) methods. The use of these methods for comparisons across multiple parameters recovered variable molecular operational taxonomic units [40,41]. These integrated approaches offer improved means of species delimitation in taxonomically difficult groups.

However, the accuracy of some species annotations has declined. For example, some sequences identified in the NCBI are misidentified as closely related species. Further, some of the results of microbial and human contamination during the experiment were mistakenly uploaded as animal barcode sequences. Some metabarcoding projects directly upload uncorrected and unadulterated comment results to the database, with many contaminated sequences of incorrect annotation and incomplete annotation. There are many sequences in the database that are annotated only to higher taxonomic levels, such as family name or genus name. These are only auxiliary references and cannot identify the species. These problems have also been found in similar studies [42,43], indicating that high-quality barcode databases need to be improved.

Species abundance: We found a high abundance of *Sarcoptes* species in this study. This may have resulted from mites parasitizing other insects that fell off, with some mites remaining on the filter membrane during the filtration process. This would have resulted in the subsequent DNA extracted by the filter membrane containing an excess of mite samples. The problem of the over-classification of microscopic insects has also been mentioned in studies using similar methods. The presence of insect fragments during the extraction procedure can lead to the over-representation of a particular taxon [44]. Filtration methods should be optimized to prevent microscopic insects from being retained on filters. In addition, the preference of PCR primers for different class groups may cause macro-barcode results that do not reflect reality [45]. The targeted optimization of primer design is required to amplify target groups [46]. Considering potential biases in sampling, as well as the processes of DNA extraction, amplification, and sequencing, it is advisable to treat the data as semi-quantitative, with the abundance of DNA reads regarded as estimates of relative abundance [47]. Metabarcoding can be convenient to provide DNA information, but to accurately determine species abundance, an improved method is still needed. PCR-free sequencing is a method that may help address these challenges [48].

### 4.4. Potential Trophic Network of Parasitoid Wasps

The DNA present in the guts of predators has been extensively documented. High-throughput sequencing methods have revealed the presence of various hexapod insects in spider guts [49], detected the dietary composition of ladybugs [50], identified prey from *Vespa mandarinia* larvae feces [51], and elucidated interactions between large mammalian herbivores and plant-dwelling arthropods [52]. Previous studies on nutritional relationships between parasitoid wasps and their hosts have primarily focused on species identification using host DNA detection techniques. For example, the quantitative aphid–parasitoid food web structure has been studied using species-specific multiplex PCR [53,54].

After the manual correction of some annotation results, we were able to eliminate classification errors and sample contamination. Dying parasitoid wasps in the ethanol traps may inadvertently consume prey DNA-contaminated liquid, but the occurrence of cross-contamination in samples stored in 95% ethanol is rare [55], so the additional insects detected in the parasitoid samples in this study appear to be mainly from parasitoid hosts. This demonstrates the feasibility of identifying host species through the DNA analysis of adult parasitoid wasps. This approach differs from the commonly used barcoding approach that relies on analyzing host samples, such as empty aphid mummies, to identify their associated parasitoid wasps [56].

## 5. Conclusions

Three main conclusions resulted from this study. (i) The high-throughput method proved to be cost-effective and capable of rapidly acquiring comprehensive DNA barcoding information from samples. (ii) The ethanol filter method efficiently captured a broad spectrum of DNA barcodes, but it had diminished resolution and reduced species richness estimates. The selective extraction of insects of a single taxonomic order enhanced the overall resolution. (iii) High-throughput sequencing on adult parasitoid wasps provided information about their hosts, facilitated understanding of host species, and improved insight into the dynamics of food webs.

## Data Availability

The data presented in the study have been deposited in the NCBI database repository under the accession number PRJNA1069984.

## Supplementary Materials

The following supporting information can be downloaded at: https://www.mdpi.com/article/10.3390/insects15040228/s1. Table S1: Alpha diversity estimates of all samples. Table S2: Taxonomy table. Table S3: Abundance in each taxa level of two methods from three locations.
